# Optimization of crystal nucleation close to a metastable fluid-fluid phase transition

**DOI:** 10.1038/srep11260

**Published:** 2015-06-22

**Authors:** Jan Wedekind, Limei Xu, Sergey V. Buldyrev, H. Eugene Stanley, David Reguera, Giancarlo Franzese

**Affiliations:** 1 Departament de Física Fonamental, Universitat de Barcelona, Martí i Franquès 1, 08028 Barcelona, Spain; 2 International Center for Quantum Materials and School of Physics, Peking University, Beijing 100871, China; 3 Department of Physics, Yeshiva University, 500 West 185th Street, New York, NY 10033 USA; 4 Center for Polymer Studies and Department of Physics, Boston University, Boston, MA 02215 USA

## Abstract

The presence of a metastable fluid-fluid critical point is thought to dramatically influence the crystallization pathway, increasing the nucleation rate by many orders of magnitude over the predictions of classical nucleation theory. We use molecular dynamics simulations to study the kinetics of crystallization in the vicinity of this metastable critical point and throughout the metastable fluid-fluid phase diagram. To quantitatively understand how the fluid-fluid phase separation affects the crystal nucleation, we evaluate accurately the kinetics and reconstruct the thermodynamic free-energy landscape of crystal formation. Contrary to expectations, we find no special advantage of the proximity of the metastable critical point on the crystallization rates. However, we find that the ultrafast formation of a dense liquid phase causes the crystallization to accelerate both near the metastable critical point and almost everywhere below the fluid-fluid spinodal line. These results unveil three different scenarios for crystallization that could guide the optimization of the process in experiments

Crystallization is a very important phenomenon that usually proceeds via the nucleation and subsequent growth of nanometer-sized crystallites that form spontaneously out of a supersaturated solution or undercooled melt^1^,^2^,^3^,^4^,^5^. This process does not occur immediately below the melting line. The formation of small crystals requires the overcoming of a free energy barrier caused by the competition between the energetic cost of maintaining the fluid-crystal interface and the free energy gained by transferring particles from the metastable fluid into the bulk crystal phase. Beyond a critical cluster size, the bulk contribution takes over and the small clusters serve as embryos for crystallization.

This initial nucleation step determines many properties of the emerging phase that are crucial in scientific and technological applications that range from material science, to medicine, or food science^2^. An extremely high nucleation barrier can prevent crystallization from occurring within any realistic time span. A low nucleation barrier, on the other hand, may produce a crystallized structure that has too many defects to be useful or that may not be thermodynamically stable. We thus need to understand the nucleation process better if we want to control it better in experiments.

This is particularly important in the case of protein crystallization because it is associated with many diseases, e.g., cataracts, sickle cell anemia, and Alzheimer’s^6^, and because high quality crystals are essential when studying the structure of proteins in diffraction experiments^7^. But many proteins are notoriously difficult to crystallize experimentally, and successful recipes for crystallization are often based on the experience and intuition of the experimenter rather than physical insight.

The main challenges in protein crystallization stem from the fact that, unlike simple molecules and atoms, proteins can be much larger than the interactions between them. As a consequence, the phase diagram of protein solutions, especially globular proteins, differ substantially from the phase diagrams of simple liquids, by having the gas-liquid critical point, and the entire gas-liquid coexistence region, below the melting line^8^,^9^. The fluid-fluid phase transition between a low protein concentration phase (“gas”) and a high protein concentration phase (“liquid”) becomes then metastable with respect to the crystal phase, but can still be observed experimentally^10^.

In recent years, simulation and theoretical studies have suggested that the presence of this metastable fluid-fluid critical point can open up new pathways for nucleation because it allows a “two-step mechanism”^11^,^12^,^13^,^14^,^15^. In this mechanism critical density fluctuations in the vicinity of the metastable critical point (i) cause a large droplet of the dense liquid to form, within which (ii) the crystal nucleation occurs. The resulting free-energy barrier to crystallization is then lowered substantially, which increases the nucleation rate by many orders of magnitude. However, recent molecular dynamics (MD) simulations conclude that this scenario does not hold true for all metastable fluid-fluid phase transitions^16^. Furthermore, experiments with globular proteins indicate that near the metastable critical point and at high supercritical protein concentrations the dynamics of the system can slow down, forming a dynamically arrested gel phase that inhibits the crystallization^17^. On the other hand, experiments with hemoglobin^18^ and polymer melts^19^ suggest that rapid crystallization is instead obtained either within the spinodal region, by a “spinodal-assisted” mechanism^19^,^20^, or by following the fluid-fluid spinodal line^18^, at least for subcritical conditions^16^.

It is thus not clear from current experimental and theoretical work whether there is a general way to optimize crystallization in systems with a metastable fluid-fluid phase transition. Here we perform a comprehensive analysis of the kinetics and thermodynamics of crystal nucleation in a wide region encompassing the metastable fluid-fluid critical point. We will show that there are three different scenarios for crystallization and that the formation of a metastable dense “liquid” patch does indeed speed up nucleation rates considerably. However, the enhancement of crystallization is associated to the whole metastable phase transition rather than to the metastable critical point itself. These results clarify the different mechanisms of crystal formation and open the door to a better experimental control and optimization of protein crystallization.

## Results

**Figure 1:**
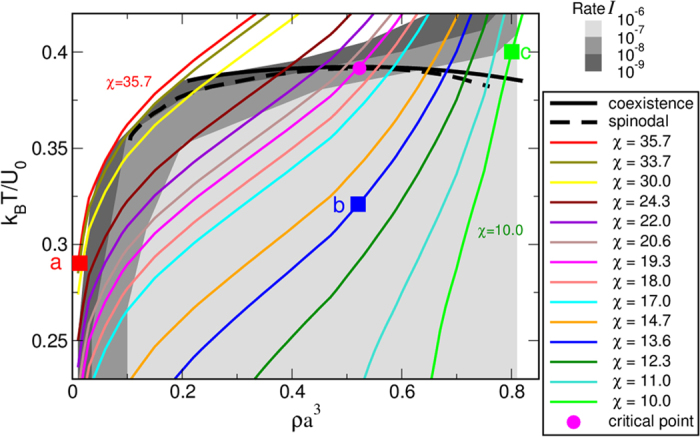
Phase diagram with iso-CNT lines. The black bold and dashed lines indicate the fluid-fluid coexistence line and spinodal line, respectively, that end at the critical point (magenta circle). The different colored lines are iso-CNT lines for different values of *χ* (as in the legend), along which simulations have been performed. The gray-shaded areas delimit regions with different values of the nucleation rates *I*, as indicated in the gray-coded scale. The red, blue, and green squares indicate the three different state points and pathways analyzed in Fig. 4.

We perform this study using MD simulations of crystal nucleation in a coarse-grained model for globular proteins with a short-range attractive interaction potential^16^


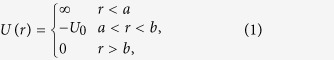


where *a* is the hard-core diameter, *b* = 1.06*a* the attractive well diameter, and *U*_0_ the attraction energy. The ratio *b*/*a* determines whether the system has a metastable liquid phase^8^,^21^,^22^, and for this choice of parameters the metastable critical point is at *T*_*c*_ = (0.3916 ± 0.0005)*U*_0_/*k*_*B*_, *ρ*_*c*_ = (0.523 ± 0.005)(1/*a*^3^), and *P*_*c*_ = (0.0519 ± 0.0005)*U*_0_/*a*^3^, with *T*_*c*_/*T*_*m*_ ≃ 0.64 at *ρ*_*c*_, where *T*_*m*_ is the temperature of sublimation^16^ (Fig. 1). This potential allows us to disentangle the effect of the dynamical arrest due to gelation-like phenomena^23^ from the effect of the slowing down at the critical point because, for this choice of parameters and for the range of densities of interest, the diffusion is only marginally affected. Thus we can explore in detail the supercritical region that was inaccessible to Muschol and Rosenberger^17^ and also the critical region^11^, the spinodal region^19^, and the subcritical region at low density^18^.

According to classical nucleation theory (CNT), the rate of crystallization *I*, defined as the number of crystals formed per unit volume and time, is given by^1^


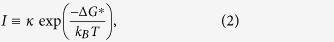


where *κ* is a kinetic pre-factor and ΔG^*^ is the nucleation barrier (i.e. the free energy for the formation of the critical cluster). Following the assumptions of Turnbull and Fisher^24^, *I* is determined by the degree of supercooling *T*_*m*_ − *T*, with a CNT nucleation barrier that is constant along the (*iso-CNT*) lines where the quantity


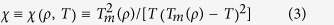


is constant^1^,^16^,^24^.

In order to analyze the effect of the fluid-fluid transition on the crystallization rate, we perform simulations along all the iso-CNT lines indicated in Fig. 1, including one located at the edge of the coexistence region (*χ* = 35.7) but that does not cross the fluid-fluid spinodal line. The details of the simulations and the methodology used to analyze the nucleation rates, the critical cluster sizes, the nucleation barriers, and the pathways for crystallization are described in the Methods section.

**Figure 2:**
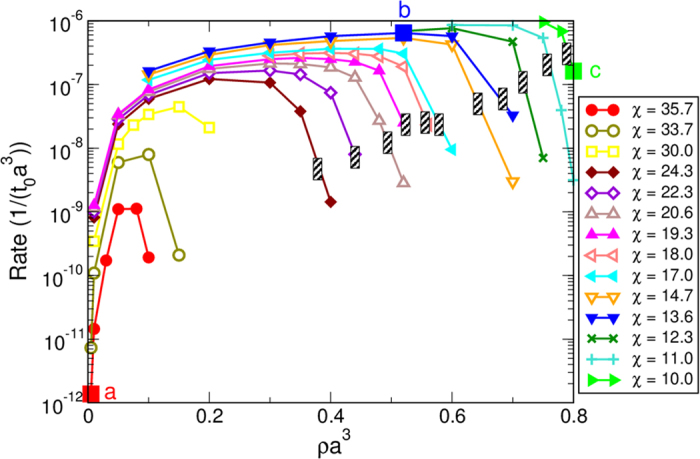
Nucleation rates versus the density for the different iso-CNT lines given in Fig. 1. The gray-dashed areas indicate the crossing of the spinodal line.

For temperatures and densities well above the liquid-liquid binodal line, the crystallization rate is so low that it cannot be observed in the simulations. However, we find that along each iso-CNT line the nucleation rate increases significantly—by more than three orders of magnitude—as we approach and cross the spinodal line, rather than staying constant as predicted by CNT (Fig. 2). Inside the spinodal region, all rates become essentially the same (within one order of magnitude), and only start to decrease significantly at very low densities. This is a strong indication that, at low densities and temperatures below the spinodal line, the crystallization becomes a process no longer controlled by the distance to the melting line *T*_*m*_, but rather by the kinetic pre-factor. The iso-CNT line at *χ* = 35.7 (red line in Figs. 1 and 2) presumably does not fully cross the spinodal line and clearly shows a different behavior: the rate, which is undetectable at high temperatures, increases quickly as we approach the spinodal line and then again drops sharply at lower temperatures and densities.

**Figure 3:**
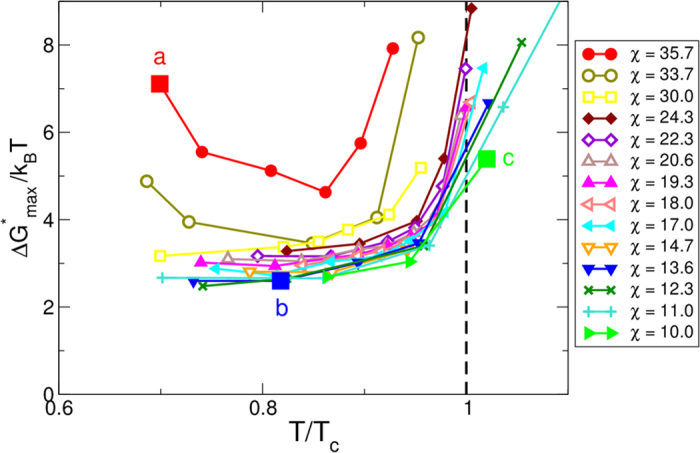
Height of the nucleation barrier for the largest crystal cluster in the system as a function of the scaled temperature *T*/*T*_*c*_ for the different iso-CNT lines.

To better understand these results, we reconstructed the free-energy landscape (Fig. 3) directly from our MD simulations using a recently developed method based on the knowledge of the mean first-passage time (MFPT), as described in Ref. 25. In agreement with the results for the nucleation rates, all curves show that the nucleation barrier drops sharply within the spinodal region. In all the cases the critical cluster size, obtained from the MFPT, is very small, typically 3–6 molecules above the spinodal line and 1–2 below the spinodal line. All the barriers estimated below the spinodal line collapse to a limiting residual value of approximately 3*k*_*B*_*T*.

There are two important conclusions that can be drawn from the figure. First, there is a clear lowering of the barrier towards crystallization that is connected to the metastable fluid-fluid transition. Inside the spinodal line, where the formation of this dense fluid phase is fast and spontaneous, the barrier towards crystallization is essentially constant, and presumably related to the residual liquid-crystal surface tension.

The second important observation is that the iso-CNT line crossing the critical point (*χ* = 19.3) does not show any special behavior with respect to the other lines crossing the spinodal line, even at densities more than 50% higher or lower than *ρ*_*c*_, at variance with what suggested earlier^11^. Thus there is no special advantage in crystallizing specifically around the critical point. Instead, what really matters is the formation of the dense liquid that occurs near and below the fluid-fluid spinodal line. This is evident from the nucleation barrier behavior corresponding to *χ* = 35.7 and 33.7, that show a minimum barrier at the point of closest approach to the spinodal line, rather than specifically at the critical point.

Consistently with these observations, we have also found that there are essentially three different pathways towards crystallization, illustrated by the three points (a,b,c) indicated by squares in Figs 1,2, and 3. They correspond to (a) crystallization at low density presumably between the binodal and the spinodal lines; (b) a point at *ρ*_*c*_ deep within the spinodal region; and (c) a location at high *ρ* outside the coexistence region.

**Figure 4:**
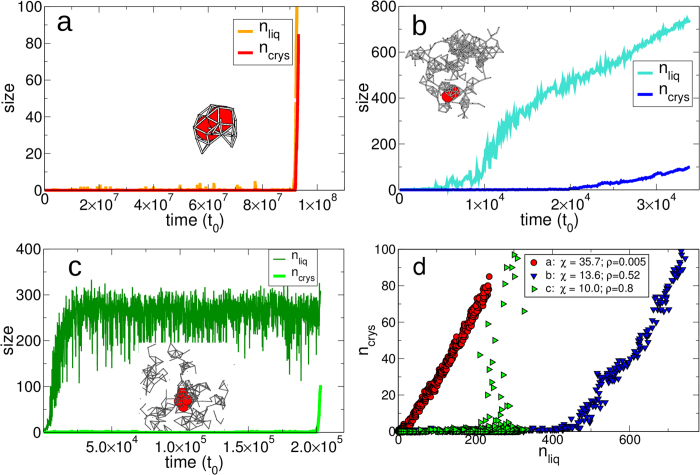
Three different crystallization mechanisms. Time evolution of the size of the largest liquid droplet and of the largest crystal obtained in a single crystallization event at (**a**) *χ* = 35.7 and *ρ* = 0.005, (**b**) *χ* = 13.6 and *ρ* = 0.52, (**c**) *χ* = 10.0 and *ρ* = 0.8. In each panel we include a snapshot of the critical cluster at the nucleation time, with crystal-like molecules in red and bonds between liquid-like molecules (not represented for clarity). (**d**) Parametric plot of the size *n*_crys_ of the crystal versus the size *n*_l*iq*_ of the liquid droplet from the previous panels.

In case (a) (Fig. 4a), for a very long time there is no dense liquid cluster forming in the system. The crystal and the liquid cluster appear simultaneously (see Fig. 4d, red circles), corresponding to nucleation of the liquid phase followed by immediate crystallization. The bottleneck for the crystallization is the formation—by spontaneous fluctuations—of a liquid-like cluster large enough to contain ~2 crystal-like molecules. Once it forms, the crystal immediately grows at the fastest possible pace and without delay. Due to the short time-scale difference between the two steps, the crystal appears directly from the vapor phase. The entire process has a very high effective free-energy barrier (Fig. 3).

In case (b), located below the spinodal line, a large liquid droplet forms *before* the crystal cluster emerges (see Fig. 4d, blue triangles). In all cases, we verify that the crystal grows inside the liquid droplet, as opposed to appearing spontaneously somewhere else in the system. Thus, the system first undergoes a rapid spinodal decomposition into gas and liquid phases (Fig. 4b). The portion of the system in the liquid phase has a density higher than the average density of the entire system at the same *T* and is at much higher supersaturation with respect to the sublimation line. As a consequence, within the liquid phase the barrier toward crystallization can be as low as 2.5*k*_*B*_*T* (Fig. 3). This residual barrier is the small, but nonetheless existing, barrier associated to the liquid-crystal surface tension. As a further proof that within the spinodal region the nucleation process is regulated by the appearance of the liquid phase, we find that the crystal nucleation rate 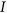 is proportional to the number of liquid-like molecules —as one would expect if crystallization is actually taking place in the liquid phase—rather than to the total number of molecules in the system, as would be the case for crystallization directly from the vapor phase.

In case (c), located above the binodal line, the dense liquid phase is unstable. However at high densities, transient liquid droplets are continuously forming and disappearing by thermal fluctuations, as indicated by the strong fluctuations in Fig. 4c. In this case, crystallization is also facilitated by the formation of dense liquid patches, but does not necessarily occur inside the largest liquid droplet (which is constantly changing) but in one with size and life-time that are large enough. This is why *n*_*crys*_ and *n*_*liq*_ seem uncorrelated in this case (Fig. 4d).

## Discussion

Our results show that there are three different scenarios for crystallization depending on where is the state point with respect to the metastable spinodal line. (a) Between the binodal and the spinodal line at subcritical conditions we observe (effectively) direct crystallization from the low-density phase. (b) Within the spinodal region we find that the pathway proceeds through a “two-step mechanism”: (i) spinodal decomposition and then (ii) nucleation-and-growth inside the high-density fluid with a very low residual barrier, much smaller than the case outside the spinodal line. Indeed, this “spinodal-assisted” mechanism could be so fast that could lead to imperfect and amorphous aggregates rather than perfect crystals. Finally, (c) above the binodal line, specially at supercritical densities, crystallization is triggered by the transient appearance of liquid droplets that are unstable but with size and life-time that are large enough to allow the stochastic formation of a crystal critical cluster. For all the pathways we find that the crystallization free-energy barriers are substantially lowered by the formation of dense liquid patches where crystallization takes place easily. This was suggested already in Ref. 11. Here we clarify that what matters is only the existence of a metastable liquid phase region as a whole, not the existence of a metastable critical point. In fact, crystallization proceeds faster below the coexistence line or even at high densities above it, than around the critical point.

We conclude that crystallization in systems exhibiting a metastable coexistence region behaves dramatically different from the prediction of CNT. In particular, the existence of a high-dense fluid phase lowers the free-energy barrier and hence accelerates the subsequent crystallization considerably; in our case by more than three orders of magnitude. This effect is not a singularity caused by the proximity of the critical point. Any state point of the system below the metastable spinodal line will give rise to a much faster crystallization because of the ultrafast formation of the dense liquid phase through spinodal decomposition. This is of particular interest for experiments that aim to leverage this effect for systems that are notoriously difficult to crystallize. Rather than focusing on just one single point, our findings suggest that there is an entire region for optimal crystallization (in dark gray in Fig. 1) that approximately follows the spinodal line at subcritical densities, overcomes the critical point and at supercritical densities moves above the binodal line. Our results indicate that, rather than trying to aim for the critical point, experiments can enhance the crystallization rate significantly at *any* density by setting *T* above the spinodal line at an appropriate distance. Even though it might seem tempting to aim for conditions within the spinodal region directly, it is more likely that crystals with many defects form under these extreme conditions. The results of our study open up many more and potentially easier-to-realize pathways for the experiments and might have a very important impact on the handcrafted art of protein crystallization.

## Methods

We use standard discrete MD simulations with a Berendsen thermostat in a cubic system with periodic boundary conditions at fixed *T* and *V*, and with *N* = 1000 molecules, a number large enough to avoid significant finite-size effects^16^,^26^. We measure temperature, *T*, in units of *U*_0_/*k*_*B*_, where *k*_*B*_ is Boltzmann constant; total volume, *V*, in units of *a*^3^; pressure, *P*, in units of *U*_0_*a*^−3^; number density *ρ* = *N*/*V* in units of *a*^−3^, and time *τ* in units of 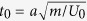, where *m* is the particle mass. For each system and set of conditions we simulate typically from 100 up to 1000 independent realizations.

We study crystallization without any bias or constraint, by rapidly quenching the fluid to a *T* well below its freezing point and following the dynamics until crystallization occurs. In the simulations, we monitor the size *n*_l*iq*_ of the largest liquid cluster, defined by molecules having at least 7 nearest neighbors (n.n.) within the attractive range (*r* < *b*), and the size *n*_c*rys*_ of the largest crystal cluster, defined by molecules having more than 11 n.n. at *r* < *b* and a local bond order parameter ≥ 0.475 as described in Ref. 16.

We obtain the crystallization rate *I* with high accuracy using the method of mean first-passage times (MFPT) *τ*_*max*_(*n*), defined as the average time at which the largest crystal cluster in the simulation reaches or exceeds the size *n* for the first time^27^. We infer the values of the critical cluster size n^*^, the Zeldovich factor *Z* and the characteristic time *τ*_*I*_ related to the nucleation rate *I* = (*τ*_*I*_*V*)^−1^, by fitting the simulation data to the function


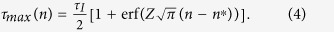


## Additional Information

**How to cite this article**: Wedekind, J. *et al*. Optimization of crystal nucleation close to a metastable fluid-fluid phase transition. *Sci. Rep*. **5**, 11260; doi: 10.1038/srep11260 (2015).
